# Associations of Family Physical Activity Support and 24-Hour Movement Behaviors with Physical Fitness in Preschool Children: A Focus on MVPA

**DOI:** 10.3390/healthcare14121668

**Published:** 2026-06-11

**Authors:** Shengyan Sun, Wenxue Sun, Shan Liao, Min Wang

**Affiliations:** 1 Faculty of Physical Education, Huzhou Normal University, Huzhou 313000, China; sysun@zjhu.edu.cn (S.S.); zjls0930@126.com (S.L.); 02148@zjhu.edu.cn (M.W.); 2 School of Electronic and Information Engineering, Huzhou College, Huzhou 313000, China

**Keywords:** preschool children, physical fitness, family physical activity support, 24-h movement behaviors

## Abstract

**Background/Objectives:** Adherence to the 24-h movement guidelines is generally low in preschool children, and less is known about how proximal family support for children’s physical activity (family PA support) is associated with physical fitness and 24-h movement behaviors. This study aimed to describe guideline adherence and to examine the associations among family PA support, 24-h movement behaviors, and physical fitness in Chinese preschool children. **Methods:** This cross-sectional study included 2386 Chinese preschool children (4.50 ± 0.86 years, 46.8% girls). Family PA support and 24-h movement behaviors were assessed using parent-reported questionnaires, and physical fitness was assessed using the Chinese National Physical Fitness Evaluation Standard for preschool children. Path analysis was used to examine the overall association pattern, including direct and indirect association estimates, among family PA support, movement behaviors, and physical fitness. **Results:** Only 12.7% of preschool children met all three 24-h movement recommendations. Compliance was 24.7% for physical activity, 82.7% for screen time, and 76.8% for sleep, indicating that insufficient physical activity was the main barrier to full guideline adherence. Family PA support was positively associated with physical fitness (β = 0.048, *p* = 0.021), and the combined indirect association estimate involving the three movement behaviors was also statistically significant (β = 0.024, *p* < 0.001). Among the three movement behaviors, family PA support was most strongly associated with higher MVPA (β = 0.150, *p* < 0.001), and MVPA showed the clearest positive association with physical fitness (β = 0.155, *p* < 0.001). Screen time was negatively associated with family PA support (β = −0.088, *p* < 0.001) but not significantly associated with physical fitness (*p* = 0.091), whereas sleep showed a small negative association with physical fitness (β = −0.056, *p* = 0.005). These findings suggest a comparatively stronger role for MVPA within the observed association pattern. **Conclusions:** Chinese preschool children showed low adherence to the 24-h movement guidelines, with insufficient physical activity appearing to be the main limiting factor. Family PA support may represent a potentially modifiable family-level correlate of preschool children’s physical fitness, with MVPA appearing to play a comparatively stronger role within the observed association pattern.

## 1. Introduction

Physical fitness is generally defined as the integrated capacity to perform daily physical activities, encompassing cardiorespiratory fitness, muscular strength and endurance, flexibility, speed, and balance [1]. In early childhood, physical fitness reflects far more than observable motor performance such as running or jumping ability; it is closely linked to multiple dimensions of health and development. Previous studies have shown that better physical fitness in preschool children is associated with more favorable body composition [2] and bone health [3], as well as enhanced cognitive development, including executive function [4] and intellectual maturity [5]. Despite its importance, physical fitness development among Chinese preschool children has not been uniformly favorable. Data from successive rounds of China’s National Physical Fitness Monitoring indicate that some fitness indicators in children aged 3–6 years have shown unfavorable trends, particularly those related to lower-limb strength, flexibility, and coordination-related performance [6,7,8]. Meanwhile, the burden of childhood overweight and obesity has continued to rise, and a global analysis identified China as one of the countries with the highest number of childhood obesity cases [9].

Among the behavioral factors relevant to physical fitness in early childhood, 24-h movement behaviors have attracted increasing attention. Within this integrated framework, physical activity (PA), screen time, and sleep are considered jointly within a finite day rather than in isolation. Contemporary 24-h movement guidelines emphasize their combined role in supporting health and development across the preschool years [10,11]. Evidence from recent studies suggests that a more favorable distribution of these behaviors is associated with a range of positive health and developmental outcomes in early childhood, including healthier body composition and cardiometabolic profiles, better psychosocial health, and improved motor development [12,13,14,15,16]. More specifically, compositional evidence from both cross-sectional and longitudinal studies suggests that allocating more time to MVPA is generally associated with more favorable physical fitness outcomes in preschool children. Cross-sectional analyses have linked greater time allocated to MVPA to better cardiorespiratory fitness, speed-agility, and muscular strength [17,18]. A recent two-year longitudinal compositional study further showed that reallocating time toward MVPA was associated with more favorable subsequent muscular strength and endurance outcomes, whereas reallocating time among sleep, sedentary behaviors, and light PA was more relevant to flexibility at follow-up [19]. Despite this evidence, adherence to the 24-h movement guidelines remains low; fewer than 15% of preschool children meet all three recommendations in China, and similarly low prevalence has been reported worldwide [20,21]. These findings suggest that it is important not only to examine how 24-h movement behaviors are associated with physical fitness, but also to identify the factors associated with variation in these behaviors during early childhood.

Preschool children’s daily movement behaviors are strongly shaped by caregivers and the home environment [22,23], making the family a key context in which PA, sedentary screen time, and sleep-related routines are established and reinforced. Existing research suggests that family influences may operate at multiple levels. Broader family-level characteristics, such as family sports attitudes, parental attitudes, and family activity choices, have been linked to children’s body mass index (BMI) [24], physical fitness, and motor development [25]. More specific home environment factors, including parental screen use, device accessibility, household rules, sleep-related parenting practices, and bedtime routines, may also shape children’s screen time and sleep [26,27]. In addition, everyday family practices such as parental support, parental modeling, parent–child play, time spent outdoors, and other home-based activity practices have been identified as relatively consistent correlates of preschool children’s PA [22,28,29]. Importantly, evidence from preschool children further suggests that, compared with parents’ own PA, what may be more directly relevant to children’s MVPA is whether broader parental orientations are translated into concrete day-to-day support practices, such as encouragement, co-activity, transportation, and other forms of practical support [30]. Taken together, these findings indicate that family influences on preschool children’s movement behaviors are multifaceted, but that proximal family PA support may be particularly relevant because it is more closely linked to children’s day-to-day movement routines and may therefore represent a more modifiable family-level factor.

Although research on preschool 24-h movement behaviors has increased in recent years, family-related studies in this area have more often focused on children’s behavior patterns or guideline adherence than on physical fitness as a downstream outcome [23,31]. Existing studies have also tended to emphasize broader family-level characteristics, such as family sports attitudes, parental education level, and family structure, or to examine family influences on isolated outcomes such as BMI, children’s PA, motor development, or selected fitness indicators rather than on physical fitness through daily movement behaviors [24,25]. As a result, it remains unclear whether proximal family support practices are associated with preschool children’s physical fitness through their day-to-day movement behaviors. This gap is especially important given that behavior patterns established in early childhood may persist over time, which may make proximal family practices a potentially meaningful target for early intervention [32].

Given the above, the present study focused on family PA support, defined as proximal family practices surrounding children’s PA, including co-participation, opportunity provision, encouragement, logistical support, and activity-related attention and communication in daily life [30,33]. By examining family PA support, 24-h movement behaviors, and physical fitness within a single path analysis model, this study extends existing research in two ways: it treats physical fitness as a downstream outcome, and it focuses on a more modifiable family-level factor than broader background characteristics. Accordingly, the present study had two aims: (1) to assess adherence to the 24-h movement guidelines in a relatively large sample of Chinese preschool children; and (2) to examine the overall association pattern among family PA support, 24-h movement behaviors, and preschool children’s physical fitness.

## 2. Materials and Methods

### 2.1. Participants

This cross-sectional study was conducted between October 2023 and December 2024. The study was approved by the Human Research Ethics Committee of Huzhou Normal University (Ref. No. 2022-09-01) and was conducted in accordance with the Declaration of Helsinki. Written informed consent was obtained from the parents or legal guardians of all participating children prior to data collection. Participants were recruited from kindergartens in four provinces of China, namely Zhejiang, Hunan, Shaanxi, and Guizhou, which were chosen from the eastern, central, northwestern, and southwestern regions of the country, respectively. These provinces were selected to enhance geographic and socioeconomic diversity in the sample, rather than to achieve national representativeness.

Within each kindergarten, children aged 3–6 years were selected using proportionate stratified random sampling based on age and sex distribution. Children were eligible if they were free from medical conditions or functional limitations that could substantially affect participation in the physical fitness assessment.

**Figure 1:**
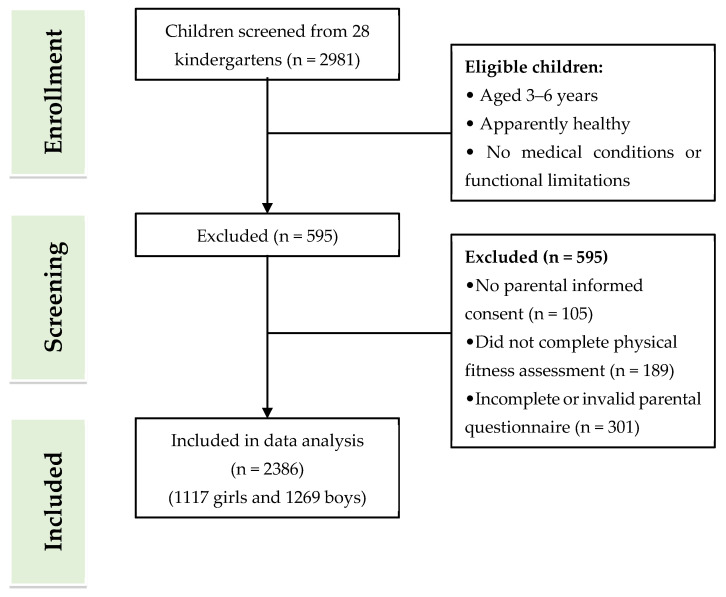
Flowchart of participant recruitment.

Prior to data collection, detailed information about the study was provided to parents. To be included in the final analytic sample, children were required to have parental consent, complete physical fitness assessment data, and valid parent questionnaire data. Of the 2981 children initially identified, 105 were excluded because informed consent was not obtained. Moreover, 189 children failed to complete the physical fitness assessment, while another 301 were excluded due to incomplete or invalid parent questionnaire data. Finally, a total of 2386 preschool children (1117 girls and 1269 boys) were included in the final analyses (Figure 1).

### 2.2. Data Collection

Data collection consisted of two components: standardized physical fitness assessments for children and parent-reported questionnaires. Physical fitness assessments were conducted in the participating kindergartens by trained assessors following a unified testing protocol. After the assessments were completed, questionnaires were distributed to the parents of tested children with the assistance of kindergarten teachers. The parent questionnaire covered three main domains: children’s background characteristics (i.e., date of birth, hereditary disease status and parental education level), 24-h movement behaviors, and family PA support. Questionnaire and physical fitness data were then matched at the individual level for subsequent analyses.

#### 2.2.1. 24-Hour Movement Behaviors

Children’s 24-h movement behaviors were assessed using parent-reported questionnaire data and classified using age-specific criteria from the Canadian 24-Hour Movement Guidelines framework in order to maintain consistency across the 3–6-year age range [10,11]. For children aged 3–4 years, adherence was defined according to the Canadian 24-Hour Movement Guidelines for the Early Years (0–4 years), namely at least 180 min/day of physical activity at any intensity, including at least 60 min/day of MVPA, no more than 60 min/day of screen time, and 10–13 h/day of sleep [10]. For children aged 5–6 years, adherence was defined according to the Canadian 24-Hour Movement Guidelines for Children and Youth (5–17 years), namely at least 60 min/day of MVPA, no more than 120 min/day of recreational screen time, and 9–11 h/day of sleep [11]. Accordingly, the overall prevalence estimate represents adherence based on age-appropriate criteria within a single integrated guideline framework.

PA was assessed using a parent-report instrument informed by the Preschool-age Physical Activity Questionnaire (Pre-PAQ) [34]. Parents were asked to report their child’s activity during the past 7 days, separately for weekdays and weekend days. Activities were classified according to the Child Activity Rating Scale (CARS) [35], which categorizes movement into five progressive intensity levels ranging from stationary (level 1) to fast or strenuous movement (level 5). Parents were instructed to estimate the duration of time their child spent at each activity level over the past week, and age-appropriate examples were provided to facilitate interpretation of the intensity categories. Mean daily duration (minutes/day) was derived using the weighted formula [(weekday × 5) + (weekend × 2)]/7. For analysis, levels 3–5 were summed to estimate TPA, and levels 4–5 were combined to derive MVPA.

Screen time was assessed by asking parents to report their child’s time spent watching television, using computers, tablets, or smartphones, or playing electronic games during the past 7 days, separately for weekdays and weekend days. Mean daily screen time was calculated as [(weekday screen time × 5) + (weekend screen time × 2)]/7.

Sleep duration was assessed using parent-reported usual bedtime, wake-up time, and daytime nap duration, recorded separately for weekdays and weekends. Nighttime sleep duration was calculated from reported bedtime and wake-up time, while nap duration was recorded independently. Average daily sleep duration was computed as [(weekday nighttime sleep × 5 + weekend nighttime sleep × 2)/7] + [(weekday nap duration × 5 + weekend nap duration × 2)/7].

#### 2.2.2. Family PA Support

Family PA support was assessed using a five-item measure developed for the present study to capture proximal family practices that support children’s PA in daily life. Item development was informed by prior research on parental and family support for children’s PA, particularly work highlighting concrete support practices such as encouragement, accompaniment, involvement, and practical or logistical support [30,33]. Accordingly, the five-item measure was designed to reflect five core support domains identified in prior literature: co-participation, opportunity provision, encouragement, logistical support, and activity-related attention and communication surrounding children’s PA.

To support content validity during the development process, the draft items were reviewed by five experts with expertise in child and adolescent physical activity, physical health promotion, and psychometrics, who rated the relevance of each item using a 4-point scale. The I-CVI values ranged from 0.80 to 1.00, and the S-CVI/Ave was 0.96, indicating good content validity. In addition, pilot testing was conducted with 67 parent respondents to assess item clarity and response feasibility, and minor wording refinements were made before the main survey was administered. The measure was originally developed in Chinese for use in the present study; therefore, no translation or cross-cultural adaptation procedure was required.

In the main survey, family PA support was assessed by asking parents to rate the extent to which they agreed with the following statements: (1) “In our family, adults often accompany the child in sports or physical activities”; (2) “In our family, adults actively arrange opportunities for the child to participate in sports or outdoor play”; (3) “In our family, adults encourage the child to be physically active in daily life”; (4) “In our family, adults provide practical support for the child’s participation in sports or physical activities”; and (5) “In our family, adults often talk about or pay attention to the child’s sports and physical activities.” Each item was rated on a 5-point Likert scale ranging from 1 (strongly disagree) to 5 (strongly agree). No items were reverse-coded. A composite score was calculated as the mean of the five items, yielding a possible score range of 1 to 5, with higher scores indicating greater family PA support. Internal consistency was assessed using Cronbach’s α and corrected item–total correlations. Construct validity was examined using confirmatory factor analysis (CFA), with standardized factor loadings, composite reliability (CR), average variance extracted (AVE), and model fit indices evaluated. Detailed item-level and scale-level psychometric results are presented in Supplementary Table S1.

#### 2.2.3. Physical Fitness Assessment

Physical fitness was assessed using the preschool section of China’s National Physical Fitness Evaluation Standard (2023 Revision). Assessments were conducted in the participating kindergartens between 9:00 and 11:30 a.m. The assessment covered anthropometric characteristics and multiple dimensions of physical performance. Specifically, anthropometric assessment included height and weight, from which body mass index (BMI) was calculated, whereas physical performance assessment included muscular strength, flexibility, coordination, speed, and balance, evaluated using handgrip strength, standing long jump, sit-and-reach, consecutive two-foot jumps, the 15 m obstacle run, and balance beam walking, respectively.

All assessments were conducted by trained examiners following the standardized protocols specified in the national standard. Each test item was scored according to age- and sex-specific normative criteria. According to the official evaluation framework, the physical fitness score was calculated as follows: Physical fitness score = 0.20 × height score + 0.10 × BMI score + 0.10 × handgrip strength score + 0.10 × standing long jump score + 0.10 × sit-and-reach score + 0.15 × consecutive two-foot jumps score + 0.10 × 15 m obstacle run score + 0.15 × balance beam walking score. The resulting composite score ranged from 0 to 100, with higher scores indicating better physical fitness.

### 2.3. Statistical Analysis

Descriptive statistics and reliability analyses were conducted using IBM SPSS Statistics (version 29.0, IBM Corp., Armonk, NY, USA), whereas confirmatory factor analysis (CFA) and path analysis were performed in R (version 4.4.1, R Foundation for Statistical Computing, Vienna, Austria) using the lavaan package (version 0.6.11). Descriptive statistics were used to summarize all study variables, with continuous variables presented as means and standard deviations and categorical variables as frequencies and percentages. Adherence to age-specific 24-h movement guidelines, including combinations of guideline adherence, was also described to characterize the behavioral profile of the sample.

Prior to structural modeling, the measurement properties of the five-item family PA support measure were evaluated. Internal consistency was assessed using Cronbach’s α and corrected item–total correlations. Construct validity was examined using CFA, with standardized factor loadings, CR, AVE, and model fit indices reported. Because the primary aim of this study was to examine the overall association structure linking family PA support, three interrelated movement behavior indicators, and physical fitness within a single analytic framework, path analysis was considered appropriate for the present study objective. The mean score of the five-item family PA support measure was therefore used as the observed indicator of family PA support in order to preserve model parsimony and interpretability. In the path model, family PA support was specified as an exogenous composite predictor, whereas MVPA, screen time, and sleep duration were modeled as interrelated behavioral variables, with physical fitness score specified as the outcome variable. Age, sex, and parental education level were included as covariates. Because the analysis focused on these three selected behavioral indicators rather than a complete 24-h time-use composition, the model was intended to characterize the overall association pattern of the variables under study rather than compositional reallocation effects.

All continuous variables were standardized prior to analysis to facilitate interpretation of standardized path coefficients. Model parameters were estimated using maximum likelihood (ML) with full information maximum likelihood (FIML) to handle missing data. To further characterize the relative prominence of different behavioral associations within the model, direct and indirect association estimates were obtained using bias-corrected bootstrap procedures with 2000 resamples [36,37]. Model fit was evaluated using multiple fit indices, including the comparative fit index (CFI), Tucker–Lewis index (TLI), root mean square error of approximation (RMSEA), and standardized root mean square residual (SRMR), following established recommendations for path analysis and structural model reporting [38,39]. Consistent with commonly applied criteria, CFI and TLI values close to or above 0.90, RMSEA values below 0.08, and SRMR values below 0.08 were interpreted as indicating acceptable model fit.

## 3. Results

### 3.1. Characteristics of the Study Population

Table 1 presents the characteristics of the study population. A total of 2386 preschool children with a mean age of 4.50 ± 0.86 years were included in the analyses, of whom 1117 (46.8%) were girls. Overall, 1258 (52.7%) of parents had attained a university education or above. On average, children accumulated 164.08 ± 65.39 min/day of TPA, including 49.31 ± 27.33 min/day of MVPA. Mean daily screen time and sleep duration were 40.84 ± 26.85 min and 11.03 ± 0.86 h/day, respectively. The mean physical fitness score was 68.05 ± 9.89.

### 3.2. Adherence to Movement Behavior Guidelines

**Figure 2:**
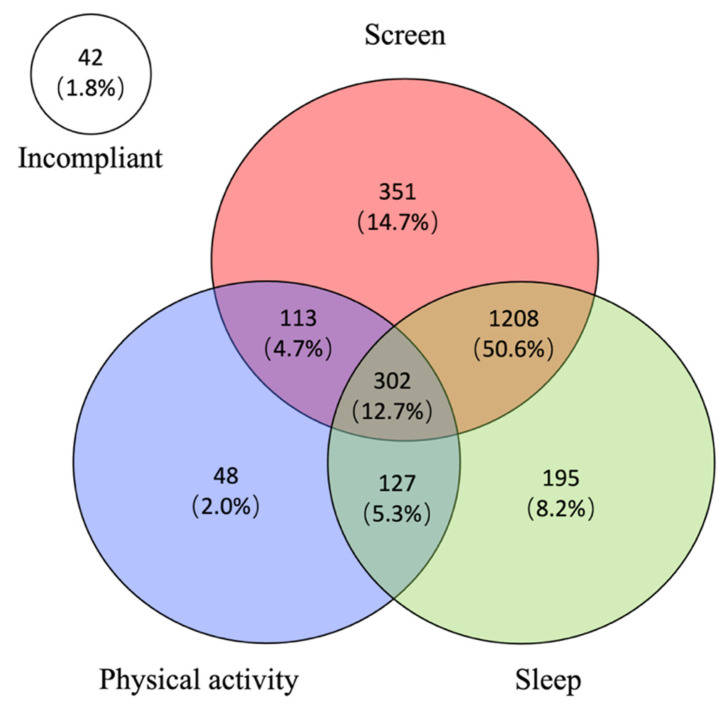
Distribution of preschool children (n = 2386) meeting the 24-h movement guidelines across physical activity, screen time, and sleep.

As shown in Figure 2, adherence was substantially higher for screen time (82.7%) and sleep (76.8%) than for PA, with only 24.7% of children meeting the PA recommendation. Overall, 12.7% of participants met all three guidelines, whereas 1.8% met none. Adherence to exactly two guidelines was common, accounting for 60.6% of the sample, and was driven primarily by the combination of sleep and screen time (50.6%). By contrast, adherence patterns involving PA were much less frequent, including PA plus sleep (5.3%) and PA plus screen time (4.7%).

### 3.3. Measurement Properties of the Family PA Support Measure

The five-item family PA support measure showed acceptable internal consistency and construct validity. Cronbach’s α was 0.845, corrected item–total correlations ranged from 0.544 to 0.635, and standardized factor loadings ranged from 0.626 to 0.909. At the scale level, composite reliability and average variance extracted were 0.820 and 0.607, respectively, and CFA indicated acceptable model fit (χ^2^ = 18.5, df = 5, *p* = 0.002, CFI = 0.937, TLI = 0.922, RMSEA = 0.065, SRMR = 0.047). Full psychometric results are provided in Supplementary Table S1.

### 3.4. Path Analysis and Association Pattern Results

As shown in Table 2, the path model showed an acceptable fit to the data (χ^2^ = 84.71, df = 17, χ^2^/df = 4.98, CFI = 0.934, TLI = 0.904, RMSEA [95% CI] = 0.041 [0.032, 0.050], and SRMR = 0.030). The model explained 7.2% of the variance in physical fitness, as well as 4.2%, 1.2%, and 5.9% of the variance in MVPA, screen time, and sleep duration, respectively.

**Figure 3:**
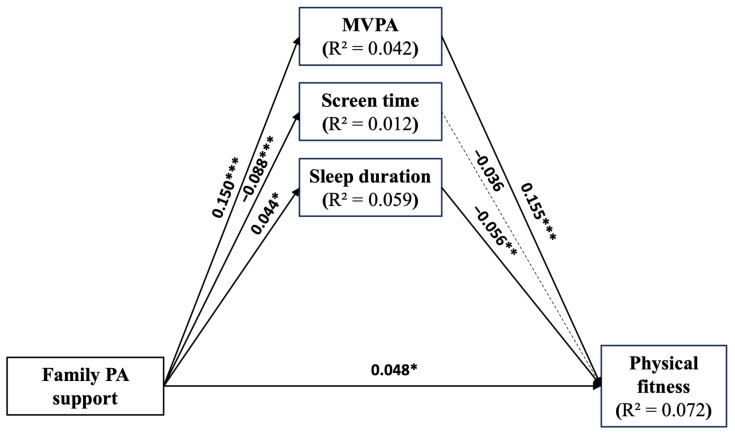
Structural model of associations among family PA support, 24-h movement behaviors, and physical fitness. Standardized path coefficients (β) are presented for all modeled associations. All displayed paths were estimated after adjustment for child age, sex, and parental education. Solid lines indicate statistically significant associations, whereas dashed lines indicate non-significant associations. Covariates were included in the model but are omitted from the figure for clarity. * *p* < 0.05, ** *p* < 0.01, *** *p* < 0.001.

Figure 3 shows the overall association pattern among family PA support, 24-h movement behaviors, and physical fitness. Family PA support was positively associated with MVPA (β = 0.150, *p* < 0.001) and sleep duration (β = 0.044, *p* = 0.030), and negatively associated with screen time (β = −0.088, *p* < 0.001). Family PA support also retained a small but significant direct association with physical fitness (β = 0.048, *p* = 0.021). Among the three movement behaviors, MVPA was positively associated with physical fitness (β = 0.155, *p* < 0.001), whereas screen time was not significantly associated with physical fitness, and sleep duration showed a small inverse association (β = −0.056, *p* = 0.005).

Detailed direct, indirect, and total association estimates are presented in Table 3. Family PA support showed a significant combined indirect association estimate with physical fitness through the three movement behaviors taken together (β = 0.024, 95% CI [0.014, 0.035], *p* < 0.001). Among the three indirect association estimates, only the MVPA-related component was statistically significant (β = 0.023, 95% CI [0.015, 0.033], *p* < 0.001), whereas the corresponding estimates involving screen time and sleep duration were not statistically significant. In addition, the total association between family PA support and physical fitness remained significant (β = 0.072, 95% CI [0.028, 0.112], *p* < 0.001). The direct effects of the covariates, including age, sex, and parental education level, were also estimated in the path model and reported in Supplementary Table S2.

## 4. Discussion

The present study extends previous research by examining how family PA support, 24-h movement behaviors, and physical fitness are linked in preschool children. Two findings are particularly noteworthy. First, overall adherence to the 24-h movement guidelines was low, and this pattern appeared to reflect particularly low compliance with the PA recommendation. Second, family PA support was associated with better physical fitness within the overall association pattern observed in the model, with MVPA showing the clearest positive association among the three movement behaviors.

The 24-h movement behavior framework emphasizes that “the whole day matters” for health promotion [40]. A growing body of research further supports this perspective, showing that more favorable combinations of physical activity, sedentary behavior, and sleep may confer synergistic benefits in early childhood [12,13,14,15,16,17]. Against this background, the present study found that only 12.7% of preschool children met all three 24-h movement recommendations. This prevalence was broadly consistent with the pooled estimate reported for Chinese preschoolers [20], and with the similarly low estimate reported for preschoolers worldwide (14.3%) [21]. More importantly, the adherence profile revealed a marked imbalance across behaviors. Although the screen time and sleep recommendations were met by 82.7% and 76.8% of children, respectively, compliance with the PA recommendation was substantially lower, with only 24.7% of children meeting this guideline. The most common combination was meeting the screen time and sleep recommendations without meeting the PA recommendation (50.6%), indicating that low overall compliance in this sample was largely attributable to low PA compliance, rather than uniform poor adherence across all three movement behaviors. A similar pattern has been observed in previous Chinese preschool studies, in which PA compliance ranged from 22.3% to 26.8%, whereas screen time and sleep compliance were markedly higher, ranging from 52.7% to 59.4% and from 89.3% to 90.7%, respectively [15,16]. Taken together, these findings indicate that PA is likely the main behavioral constraint in achieving a balanced 24-h movement profile among preschool children. This suggests that PA may warrant particular attention in early intervention efforts.

Given this guideline adherence profile, it is particularly important to consider how family PA support may be associated with preschool children’s daily movement patterns. In the present study, higher family PA support was associated with more MVPA, less screen time, and slightly longer sleep duration. This overall pattern suggests that family support may be closely linked to the organization of children’s daily routines. This interpretation can be understood within Bronfenbrenner’s ecological systems theory, which positions the family as the most immediate microsystem influencing young children’s development through everyday interactions and structured routines [41]. Among the three behavioral pathways, the association between family PA support and MVPA was the most clearly observed. This is plausible given that concrete parental support practices, such as co-participation, encouragement, logistical support, modeling, and the provision of time and opportunities for activity, may be associated with greater opportunities for children’s active play and therefore with higher MVPA in daily life [30,33,42,43]. Notably, direct evidence linking specific parental support dimensions to children’s PA or MVPA has been reported more often in school-aged samples than in preschool children. The present findings therefore add to the still limited preschool evidence on this issue. The negative association between family PA support and screen time observed in our study may reflect the fact that families who actively support children’s PA are more likely to offer activity alternatives and to regulate screen-related routines through household practices and parental monitoring [27]. By contrast, there was a small positive association between family PA support and sleep. One possible explanation is that family PA support may partly overlap with broader family routines, as sleep-related parenting practices and consistent bedtime routines have been linked to better sleep outcomes in children [26].

Among the 24-h movement behaviors, MVPA showed the clearest positive association with physical fitness (β = 0.155, *p* < 0.001), suggesting that higher-intensity active play may play a comparatively more relevant role than the other two behaviors in relation to physical fitness in preschool children. This finding is consistent with compositional and isotemporal substitution studies showing that reallocating time toward MVPA is associated with more favorable performance in cardiorespiratory fitness, muscular strength, and speed-agility, as well as with subsequent improvements in selected fitness components [17,18,19]. Such an association is biologically plausible, as MVPA typically imposes greater cardiometabolic and neuromuscular demands than lower-intensity activity, involving more frequent running, jumping, accelerating, and direction-changing movements that may provide stronger stimuli for fitness development in early childhood [44]. Screen time showed a different pattern. Previous evidence in children and adolescents has been mixed, with one study reporting no significant association with physical fitness [45], and another reporting inverse associations [46]. One possible explanation for these differing findings is variation in study populations and screen exposure profiles. In the latter study, the sample consisted of school-aged children and adolescents with a mean screen time of 9.1 h/day and generally low screen time guideline adherence, suggesting both higher screen exposure and greater variability, which may make inverse associations with physical fitness easier to detect [46]. In the present preschool sample, mean screen time was much lower (40.84 ± 26.85 min/day), and 82.7% of children met the recommendation. This relatively low level of screen exposure, together with the high rate of guideline adherence, may have limited the variability of screen time and reduced its discriminatory power in the present model. In addition, because screen time represents only one component of sedentary behavior, its unique association may be attenuated when other co-occurring movement behaviors are considered simultaneously within the 24-h time-use structure [47,48]. A more cautious interpretation is warranted for the inverse association observed between sleep duration and physical fitness. Although sleep duration was negatively associated with physical fitness in the present model, the effect was small and should not be taken as evidence that longer sleep itself is unfavorable for physical fitness. One possible explanation is that, in preschool children, longer total sleep duration may partly reflect age-related sleep needs and greater daytime napping, because younger children typically sleep longer and nap more during the day, but may also perform less well on physical fitness tests than older preschoolers. This age-related pattern could therefore contribute to the observed inverse association. It is also possible that allocating more time to sleep leaves less time available for active movement within a fixed 24-h day, given that movement behaviors are co-dependent [47]. Moreover, previous evidence suggests that both insufficient and excessive sleep may be associated with unfavorable physical fitness outcomes [49], which implies that the sleep–fitness relationship may be non-linear rather than consistently beneficial across the full range of sleep duration. Because only total sleep duration was assessed, the observed inverse association may also partly reflect unmeasured differences in sleep pattern or daily routines, rather than a detrimental effect of longer sleep itself. Taken together, these behavior-specific findings suggest that the fitness relevance of 24-h movement behaviors is not evenly distributed across components, with MVPA showing the clearest positive association in the present model.

Overall, the association pattern observed in the present study indicates that family PA support was more closely associated with physical fitness through children’s MVPA than through the other two 24-h movement behaviors. The significant indirect association was primarily reflected in the MVPA-related component, which is consistent with earlier preschool evidence showing that family support-related practices may represent a more proximal correlate of child MVPA than broader parental factors [30]. This suggests that family PA support may be particularly relevant to children’s day-to-day movement routines. At the same time, the persistence of a significant direct association, together with the modest explained variance of the model (i.e., R^2^ = 0.042 for MVPA and R^2^ = 0.072 for physical fitness), suggests that additional factors not captured by the present model may also contribute to preschool children’s physical fitness, such as activity-related experiences, socioeconomic circumstances, home or neighborhood environments, genetic predispositions, and other child-level developmental characteristics [25,50,51]. Because studies directly examining how proximal family support, daily movement behaviors, and physical fitness are jointly associated remain scarce in preschool populations, the present findings add preschool-specific evidence that MVPA may play a comparatively stronger role in this overall association pattern.

From a practical perspective, the present findings suggest that family PA support may represent a potentially modifiable family-level factor relevant to preschool children’s physical fitness within the observed associations. In particular, the results highlight MVPA as a comparatively important behavioral target, supporting the potential value of family-based strategies that promote co-participation, activity opportunities, encouragement, and practical support. In the context of generally low compliance with the PA recommendation, interventions that help families create more opportunities for higher-intensity active play may be particularly valuable. However, these practical implications should be interpreted cautiously, as the observed patterns are associative rather than causal and require further confirmation in longitudinal and intervention studies.

However, several limitations should be considered when interpreting the present findings. First, PA, screen time, and sleep duration were assessed using parent-reported questionnaires rather than objective monitoring devices, which may have introduced recall and social desirability bias, particularly in the estimation of higher-intensity activity such as MVPA. Future studies would benefit from objective measurement approaches such as accelerometry. Second, because the path model was based on cross-sectional data, the observed direct and indirect associations should be interpreted as patterns of association rather than evidence of temporal ordering, causality, or a confirmed behavioral mechanism. Longitudinal and intervention-based designs are needed to strengthen causal inference. In addition, because the present study modeled three selected behavioral indicators rather than a complete 24-h time-use composition, future studies using complete daily time-use data may benefit from compositional data analysis to more explicitly examine the co-dependent structure of movement behaviors and potential time-reallocation effects. Third, the five-item family PA support measure was developed for this study and has not yet been validated in independent samples or different cultural contexts, which may limit the generalizability of the findings and their comparability with previous research. Fourth, although the sample included children from four provinces, it was limited to kindergarten-attending children and was not nationally representative. This may have excluded certain subgroups and limited the generalizability of the findings. Future research in more diverse and nationally distributed samples would help extend the generalizability of the evidence.

## 5. Conclusions

This study examined 24-h movement guideline adherence and the associations among family PA support, 24-h movement behaviors, and physical fitness in preschool children. Overall, adherence to the 24-h movement guidelines was low in this sample of Chinese preschool children, with insufficient PA emerging as the main behavioral shortfall rather than uniformly poor compliance across all three behaviors. Family PA support was positively associated with preschool children’s physical fitness, and this association appeared to be mainly reflected through children’s daily MVPA. Among the three movement behaviors, MVPA showed the clearest positive association with physical fitness, whereas screen time and sleep showed weaker associations. Taken together, these findings suggest that strengthening family support for children’s PA may be a relevant family-level strategy for promoting physical fitness in preschool children, particularly by supporting greater engagement in MVPA.

## Figures and Tables

**Table 1:** Descriptive characteristics of the sample.

Variable	n (%)	Mean (SD)
Age (yrs)		4.50 (0.86)
Gender	2386	
Girls	1117 (46.8%)	
Boys	1269 (53.2%)	
Height (cm)		107.15 (7.45)
Weight (kg)		17.67 (3.36)
BMI (kg/m^2^)		15.28 (1.56)
Parental education level		
Below university	1128 (47.3%)	
University or higher	1258 (52.7%)	
MVPA (min/d)		49.31 (27.33)
TPA (min/d)		164.08 (65.39)
Screen time (min/d)		40.84 (26.85)
Sleep duration (h/d)		11.03 (0.86)
Physical fitness score		68.05 (9.89)

Note. BMI, body mass index; MVPA, moderate-to-vigorous physical activity; TPA, total physical activity.

**Table 2:** Fit indices for the path model.

Metric	χ^2^	df	χ^2^/df	CFI	TLI	RMSEA	RMSEA 95% CI	SRMR
Value	84.71	17	4.98	0.934	0.904	0.041	[0.032, 0.050]	0.030
Criterion	—	—	<5.00	>0.900	>0.900	<0.080	—	<0.080

**Table 3:** Direct, indirect, and total association estimates in the structural model.

Path	β (std.)	95% CI	*p*
**Direct association estimates**			
Family PA support → MVPA	0.150	[0.111, 0.190]	<0.001
Family PA support → Screen time	−0.088	[−0.127, −0.046]	<0.001
Family PA support → Sleep duration	0.044	[0.004, 0.081]	0.030
MVPA → Physical fitness	0.155	[0.115, 0.194]	<0.001
Screen time → Physical fitness	−0.036	[−0.077, 0.006]	0.091
Sleep duration → Physical fitness	−0.056	[−0.096, −0.019]	0.005
Family PA support → Physical fitness	0.048	[0.006, 0.087]	0.021
**Behavior-specific indirect association estimates**			
Family PA support → MVPA → Physical fitness	0.023	[0.015, 0.033]	<0.001
Family PA support → Screen time → Physical fitness	0.003	[0.000, 0.008]	0.148
Family PA support → Sleep duration → Physical fitness	−0.002	[−0.006, 0.000]	0.088
**Combined indirect association estimate**			
Family PA support → 24 HMBs → Physical fitness	0.024	[0.014, 0.035]	<0.001
**Total association estimate**			
Family PA support → Physical fitness	0.072	[0.028, 0.112]	<0.001

Note. 24HMBs = 24-h movement behaviors. Estimates are standardized coefficients (β) with 95% bias-corrected bootstrap confidence intervals based on 2000 resamples. All *p*-values are two-tailed. The arrow symbol (→) indicates the association pathway specified in the path model and does not imply causality.

## Data Availability

Data are not publicly available due to privacy and ethical restrictions involving preschool children but are available from the corresponding author on reasonable request.

## Supplementary Materials

The following supporting information can be downloaded at: https://www.mdpi.com/article/10.3390/healthcare14121668/s1, Table S1: Reliability, validity, and CFA model fit results for the family PA support measure; Table S2: Direct association estimates for covariates in the structural model.

## References

[1] Ortega F.B., Ruiz J.R., Castillo M.J., Sjöström M. (2008). Physical fitness in childhood and adolescence: A powerful marker of health. Int. J. Obes..

[2] Henriksson P., Leppänen M.H., Henriksson H., Delisle Nyström C., Cadenas-Sanchez C., Ek A., Ruiz J.R., Ortega F.B., Löf M. (2019). Physical fitness in relation to later body composition in pre-school children. J. Sci. Med. Sport.

[3] Gómez-Bruton A., Marín-Puyalto J., Muñiz-Pardos B., Lozano-Berges G., Cadenas-Sanchez C., Matute-Llorente A., Gómez-Cabello A., Moreno L.A., Gonzalez-Agüero A., Casajus J.A. (2020). Association between physical fitness and bone strength and structure in 3- to 5-year-old children. Sports Health.

[4] Zhou Z., Chen Y., Huang K., Zeng F., Liang Z., Wang N., Chen Z., Deng C. (2024). Relationship between physical fitness and executive function in preschool children: A cross-sectional study. BMC Sports Sci. Med. Rehabil..

[5] Latorre-Román P.Á., Mora-López D., García-Pinillos F. (2016). Intellectual maturity and physical fitness in preschool children. Pediatr. Int..

[6] General Administration of Sport of China 2010 National Physical Fitness Monitoring Bulletin. https://www.sport.gov.cn/n315/n329/c965571/content.html.

[7] General Administration of Sport of China 2014 National Physical Fitness Monitoring Bulletin. https://www.sport.gov.cn/n315/n329/c216784/content.html.

[8] General Administration of Sport of China Release of the Fifth National Physical Fitness Monitoring Bulletin. https://www.sport.gov.cn/n315/n329/c24335066/content.html.

[9] The GBD 2015 Obesity Collaborators (2017). Health effects of overweight and obesity in 195 countries over 25 years. N. Engl. J. Med..

[10] Tremblay M.S., Chaput J.-P., Adamo K.B., Aubert S., Barnes J.D., Choquette L., Duggan M., Faulkner G., Goldfield G.S., Gray C.E. (2017). Canadian 24-hour movement guidelines for the early years (0–4 years): An integration of physical activity, sedentary behavior, and sleep. BMC Public Health.

[11] Tremblay M.S., Carson V., Chaput J.-P., Connor Gorber S., Dinh T., Duggan M., Faulkner G., Gray C.E., Gruber R., Janson K. (2016). Canadian 24-hour movement guidelines for children and youth: An integration of physical activity, sedentary behavior, and sleep. Appl. Physiol. Nutr. Metab..

[12] Mavilidi M.F., Zou L., Li J., Cliff D.P., Pesce C., Abdeta C., Paas F., Howard S.J. (2025). Adherence to 24-hour movement guidelines: Cognitive effects in Australian preschoolers. Ment. Health Phys. Act..

[13] Ding H., Jiang L., Seo H., Chun B. (2025). Association between meeting the 24-hour movement guidelines and cardiometabolic risk factors in toddlers, preschoolers, children, and adolescents: A systematic review and meta-analysis. J. Sports Sci..

[14] Feng J., Zheng C., Sit C.H.-P., Reilly J.J., Huang W.Y. (2021). Associations between meeting 24-hour movement guidelines and health in the early years: A systematic review and meta-analysis. J. Sports Sci..

[15] Li F., Yin L., Sun M., Gao Z. (2022). Examining relationships among Chinese preschool children’s meeting 24-hour movement guidelines and fundamental movement skills. J. Clin. Med..

[16] Yin L., Li F., Liu P., Yin Z., Yang Z., Pi L., Gao Z. (2024). Examining the relationship between meeting 24-hour movement behavior guidelines and mental health in Chinese preschool children. Front. Pediatr..

[17] Lemos L., Clark C., Brand C., Pessoa M.L., Gaya A., Mota J., Duncan M., Martins C. (2021). 24-hour movement behaviors and fitness in preschoolers: A compositional and isotemporal reallocation analysis. Scand. J. Med. Sci. Sports.

[18] Lu Z., Guo J., Liu C., Wu J., Zhao C., Wang F., Bao Y., Zhang H., Qi B., Li X. (2024). Reallocation of time to moderate-to-vigorous physical activity and estimated changes in physical fitness among preschoolers: A compositional data analysis. BMC Public Health.

[19] Song H., Zhang B., Lu N., Liu Y., Lau P.W.C. (2025). Longitudinal associations between 24-hour movement behaviors and physical fitness in preschoolers: A compositional isotemporal substitution analysis. Sci. Rep..

[20] Zhang Z., Li R., Xie Y., Chen Z., Bailey R., Khoo S. (2025). Prevalence of meeting 24-hour movement guidelines in China: A systematic review and meta-analysis. BMC Public Health.

[21] Chong K.H., Suesse T., Cross P.L., Ryan S.T., Aadland E., Aoko O., Byambaa A., Carson V., Chaput J.-P., Christian H. (2024). Pooled analysis of physical activity, sedentary behavior, and sleep among children from 33 countries. JAMA Pediatr..

[22] Lindsay A.C., Greaney M.L., Wallington S.F., Mesa T., Salas C.F. (2017). A review of early influences on physical activity and sedentary behaviors of preschool-age children in high-income countries. J. Spec. Pediatr. Nurs..

[23] Dugger R., Williams T., Burkart S., Zhu X.X., Reesor-Oyer L., Pfledderer C.D., von Klinggraeff L., Parker H., White J., McLain A.C. (2025). Family and home environment predictors of children’s 24-hour movement guideline adherence: A mixed-methods study. Child. Obes..

[24] Yin L., Chen P., Wang K., Zhang T., Liu H., Yang J., Lu W., Luo J. (2021). The influence of family sports attitude on children’s sports participation, screen time, and body mass index. Front. Psychol..

[25] Hu B.Y., Wu Z., Kong Z. (2022). Family physical activities choice, parental views of physical activities, and Chinese preschool children’s physical fitness and motor development. Early Child. Educ. J..

[26] Cook G., Carter B., Wiggs L., Southam S. (2024). Parental sleep-related practices and sleep in children aged 1–3 years: A systematic review. J. Sleep Res..

[27] Veldman S.L.C., Altenburg T.M., Chinapaw M.J.M., Gubbels J.S. (2023). Correlates of screen time in the early years (0–5 years): A systematic review. Prev. Med. Rep..

[28] Yao C.A., Rhodes R.E. (2015). Parental correlates in child and adolescent physical activity: A meta-analysis. Int. J. Behav. Nutr. Phys. Act..

[29] Bingham D.D., Costa S., Hinkley T., Shire K.A., Clemes S.A., Barber S.E. (2016). Physical activity during the early years: A systematic review of correlates and determinants. Am. J. Prev. Med..

[30] Dowda M., Pfeiffer K.A., Brown W.H., Mitchell J.A., Byun W., Pate R.R. (2011). Parental and environmental correlates of physical activity of children attending preschool. Arch. Pediatr. Adolesc. Med..

[31] Feng J., Huang W.Y., Sit C.H.-P., Reilly J.J., Khan A. (2024). Effectiveness of a parent-focused intervention targeting 24-hour movement behaviors in preschool-aged children: A randomised controlled trial. Int. J. Behav. Nutr. Phys. Act..

[32] Lioret S., Campbell K.J., McNaughton S.A., Cameron A.J., Salmon J., Abbott G., Hesketh K.D. (2020). Lifestyle patterns begin in early childhood, persist and are socioeconomically patterned, confirming the importance of early life interventions. Nutrients.

[33] Liu Y., Zhang Y., Chen S., Zhang J., Guo Z., Chen P. (2017). Associations between parental support for physical activity and moderate-to-vigorous physical activity among Chinese school children: A cross-sectional study. J. Sport Health Sci..

[34] Dwyer G.M., Hardy L.L., Peat J.K., Baur L.A. (2011). The validity and reliability of a home environment preschool-age physical activity questionnaire (Pre-PAQ). Int. J. Behav. Nutr. Phys. Act..

[35] Puhl J., Greaves K., Hoyt M., Baranowski T. (1990). Children’s activity rating scale (CARS): Description and calibration. Res. Q. Exerc. Sport.

[36] MacKinnon D.P., Lockwood C.M., Williams J. (2004). Confidence limits for the indirect effect: Distribution of the product and resampling methods. Multivar. Behav. Res..

[37] Preacher K.J., Hayes A.F. (2008). Asymptotic and resampling strategies for assessing and comparing indirect effects in multiple mediator models. Behav. Res. Methods.

[38] Hu L., Bentler P.M. (1999). Cutoff criteria for fit indexes in covariance structure analysis: Conventional criteria versus new alternatives. Struct. Equ. Model..

[39] Kline R.B. (2023). Principles and Practice of Structural Equation Modeling.

[40] Rollo S., Antsygina O., Tremblay M.S. (2020). The whole day matters: Understanding 24-hour movement guideline adherence and relationships with health indicators across the lifespan. J. Sport Health Sci..

[41] Bronfenbrenner U. (1979). The Ecology of Human Development: Experiments by Nature and Design.

[42] An M., Chen T., Zhou Q., Ma J. (2021). Paternal and maternal support of moderate-to-vigorous physical activity in children on weekdays and weekends: A cross-sectional study. BMC Public Health.

[43] Lijuan W., Jiancui S., Suzhe Z. (2017). Parental influence on the physical activity of Chinese children: Do gender differences occur?. Eur. Phys. Educ. Rev..

[44] Alpert B., Field T., Goldstein S., Perry S. (1990). Aerobics enhances cardiovascular fitness and agility in preschoolers. Health Psychol..

[45] Chen Z., Chi G., Wang L., Chen S., Yan J., Li S. (2022). The combinations of physical activity, screen time, and sleep, and their associations with self-reported physical fitness in children and adolescents. Int. J. Environ. Res. Public Health.

[46] Dong Q., Liu H., Fu Q. (2025). Associations between physical fitness and different aspects of screen time in children and adolescents. Sci. Rep..

[47] Dumuid D., Stanford T.E., Martin-Fernández J.-A., Pedišić Ž., Maher C.A., Lewis L.K., Hron K., Katzmarzyk P.T., Chaput J.-P., Fogelholm M. (2018). Compositional data analysis for physical activity, sedentary time and sleep research. Stat. Methods Med. Res..

[48] Pedišić Ž., Dumuid D., Olds T.S. (2017). Integrating sleep, sedentary behavior, and physical activity research in the emerging field of time-use epidemiology: Definitions, concepts, statistical methods, theoretical framework, and future directions. Kinesiology.

[49] Xiong X., Cui Y., Zhang W., Zhao C., Wu J., Li H., Zhen Z., Sun J. (2022). Association between sleep duration and physical fitness in children aged 3–6 years: A cross-sectional study from China. Int. J. Environ. Res. Public Health.

[50] Chen J., Song W., Zhao X., Lou H., Luo D. (2023). The relationship between fundamental motor skills and physical fitness in preschoolers: A short-term longitudinal study. Front. Psychol..

[51] Figueroa R., An R. (2017). Motor skill competence and physical activity in preschoolers: A review. Matern. Child Health J..

