# Supplementary material for: Associations of Family Physical Activity Support and 24-Hour Movement Behaviors with Physical Fitness in Preschool Children: A Focus on MVPA
Source: Healthcare (Basel). 2026 Jun 11;14(12):1668. doi: 10.3390/healthcare14121668 (PMC13300133; doi:10.3390/healthcare14121668)
Supplement: Supplementary file 1 [file healthcare-14-01668-s001.zip › healthcare-4292577-supplementary.pdf]

Table S1. Reliability, validity, and CFA model fit results for the family PA support measure

Panel A. Item-level results

No.	Item statement	Corrected item–total correlation	Standardized loading
1	In our family, adults often accompany the child in sports or physical activities.	0.589	0.777
2	In our family, adults actively arrange opportunities for the child to participate in sports or outdoor play.	0.593	0.909
3	In our family, adults encourage the child to be physically active in daily life.	0.544	0.626
4	In our family, adults provide practical support for the child's participation in sports or physical activities (e.g., accompanying, transportation, arranging opportunities).	0.635	0.814
5	In our family, adults often talk about or pay attention to the child's sports and physical activities.	0.601	0.679

Panel B. Scale-level results

Items	Cronbach's α	CR	AVE
5	0.845	0.820	0.607

Panel C. CFA model fit indices

χ^2	df	p	CFI	TLI	RMSEA	SRMR
18.5	5	0.002	0.937	0.922	0.065	0.047

Note. Corrected item–total correlation and standardized factor loading are reported at the item level. Cronbach's α, composite reliability (CR), and average variance extracted (AVE) are reported for the five-item measure as a whole. CFA model fit indices are reported for the overall measurement model.

Table S2. Direct association estimates for covariates in the structural model

Path	β (std.)	95% CI	p
Age → MVPA	-0.014	[-0.053, 0.023]	0.477
Sex → MVPA	0.002	[-0.038, 0.042]	0.928
Parental education → MVPA	-0.011	[-0.053, 0.029]	0.592
Age → Screen time	0.028	[-0.014, 0.068]	0.178
Sex → Screen time	-0.002	[-0.043, 0.038]	0.924
Parental education → Screen time	-0.003	[-0.043, 0.037]	0.901
Age → Sleep duration	-0.231	[-0.269, -0.191]	<0.001
Sex → Sleep duration	-0.047	[-0.086, -0.009]	0.019
Parental education → Sleep duration	-0.064	[-0.104, -0.025]	0.002
Age → Physical fitness	0.180	[0.140, 0.219]	<0.001
Sex → Physical fitness	0.005	[-0.034, 0.043]	0.802
Parental education → Physical fitness	-0.042	[-0.081, -0.003]	0.033

Note. Estimates are standardized coefficients (β) with 95% bias-corrected bootstrap confidence intervals based on 2,000 resamples. Sex was coded as 0 = girls and 1 = boys. Parental education was treated as an ordinal variable, with higher values indicating higher educational attainment. All p-values are two-tailed.
